# Incision of the Vulva as a Preventive Measure against Ruptured Perinœom

**Published:** 1852-01

**Authors:** 


*Incision of the Vulva as a preventive measure against ruptured Perinœum.* By M. M. Paul Dubois and Chailly-Honore. (London Lancet, April 19, 1851.) M. Paul Dubois, and lately M. Chailly Honore, strongly advocate an oblique incision of about a third of an inch on the vulva by the side of the perinœum, either to prevent altogether the rupture of that region when much distended, or when the rupture is inevitable, to promote it on a spot where it can produce but little mischief. Both the above accoucheurs support their views by successful cases.—*Charleston (S. C.) Med. Jour.*

